# Common Variant of *FTO* Gene, rs9939609, and Obesity in Pakistani Females

**DOI:** 10.1155/2013/324093

**Published:** 2013-09-02

**Authors:** Adeela Shahid, Sobia Rana, Shahid Saeed, Muhammad Imran, Nasir Afzal, Saqib Mahmood

**Affiliations:** ^1^Department of Physiology and Cell Biology, University of Health Sciences, Khayaban-e-Jamia Punjab, Lahore-54600, Pakistan; ^2^ Department of Physiology, Shalamar Medical and Dental College, Shalimar Link Road, Mughalpura, Lahore-54840, Pakistan; ^3^Department of Physiology, King Edward Medical University, Nelagumbad, Anarkali, Lahore-54000, Pakistan; ^4^Department of Physiology, College of Medicine, University of Dammam, Dammam 34100, Saudi Arabia; ^5^Department of Human Genetics & Molecular Biology, University of Health Sciences, Khayaban-e-Jamia Punjab, Lahore-54600, Pakistan

## Abstract

Numerous studies confirmed the association of *FTO* (fat mass and obesity associated gene) common variant, rs9939609, with obesity in European populations. However, studies in Asian populations revealed conflicting results. We examined the association of rs9939609 variant of *FTO* gene with obesity and obesity-related anthropometric and metabolic parameters in Pakistani population. Body weight, height, waist circumference, hip circumference, and blood pressure (BP) were measured. BMI and waist-to-hip ratio (WHR) were calculated. Levels of fasting blood glucose (FBG), insulin, leptin, and leptin receptors were measured by enzyme linked immunosorbent assay (ELISA), and homeostasis model assessment of insulin resistance (HOMA-IR) was calculated. The results showed association of *FTO* gene, rs9939609, with obesity in females (>18 years of age). *FTO* minor allele increased the risk of obesity by 2.8 times (95% CI = 1.3–6.0) in females. This allele showed association with body weight, BMI, waist circumference, hip circumference, WHR, BP, plasma FBG levels, HOMA-IR, plasma insulin levels, and plasma leptin levels. In conclusion, *FTO* gene, rs9939609, is associated with BMI and risk of obesity in adult Pakistani females. Association of rs9939609 variant with higher FBG, plasma insulin, and leptin levels indicates that this polymorphism may disturb the metabolism in adult females and predispose them to obesity and type 2 diabetes. However, the above-mentioned findings were not seen in children or males.

## 1. Introduction

Obesity is a serious public health problem of 21st century not only in adults but also among children and adolescents. It is associated with various adverse health outcomes such as type 2 diabetes (T2D), hypertension, stroke, cardiovascular disease (CVD), cancer, and early deaths. It represents a major epidemic not only in western countries but also in South East Asia. According to WHO, National Health Survey (NHS 1990–1994) reported 5.2% females and 1.6% males in Pakistan above the age of 15 years were obese in Pakistan [1]. 

Obesity is a multifactorial and heterogeneous condition due to complex interaction of genetic, behavioral, developmental, and environmental factors [2, 3]. Genetic factors are responsible for 40%–70% of variation in human obesity [4]. Multiple allelic variants interact with one another and with environmental factors resulting in expression of obesity and its associated phenotypes [5]. Many allelic variants of fat mass and obesity associated (*FTO*) gene have been linked with BMI and obesity [6–8]. A common variant (rs9939609) in the first intron of *FTO* gene with T to A change has been associated with T2D through its effect on BMI. The individuals homozygous for this particular variant (allele A) had a higher BMI as compared to heterozygous individuals. The risk of high BMI and predisposition to diabetes was additive [6]. 

The association of *FTO* rs9939609 variant with obesity has been replicated in different European populations and strongly pointed the association of this variant with diabetes [9–13]. On the other hand, studies in Chinese Han [14], Oceanic [15], and African populations [16] failed to confirm rs9939609 variant as a major contributor to obesity and T2D. Studies in Korean, Malay, Japanese, Canadians of South Asian origin, and Chinese populations have reported the association of this SNP with BMI and obesity [17–23]. Despite less frequency of risk allele A in Chinese population, its effect on BMI was the same as that in the European population [17]. *FTO* variant associated strongly with T2D but weakly with obesity in Indians [24]. In short, the disease association of the *FTO* variant varies with ethnicity and populations.

Gender association of rs9939609 variant with obesity and BMI in girls is also observed [25]. Association of rs9939609 variant with obesity decreases with age [26]. *FTO *variant has less effect in old age as compared to young ones [27]. Recently, a study reported the association of rs9939609 variant with high leptin levels suggesting the role of this SNP in inducing leptin resistance and disturbing the regulation of food intake and energy expenditure leading to obesity [28].

*FTO *variant has been studied in various Asian populations for its association with obesity, but the results of these studies are controversial in these populations. Few studies have been carried out in population regarding the association of *FTO* gene variant, rs9939609, with obesity in South Asia. A dramatic increase in the frequency of obesity has been seen in Pakistan [29]. In the present study, rs9939609 variant has been genotyped in Pakistani obese and nonobese children, adolescents, and adults to investigate association of this variant with obesity and obesity-related anthropometric and metabolic traits. 

## 2. Material and Methods

### 2.1. Subjects

The study was carried out at University of Health Sciences, Lahore, Pakistan. Obese and nonobese subjects (*n* = 394, age range 5–45 years) were recruited from local, public, and private hospitals, schools, colleges, and universities after obtaining informed consent. Simple random sampling without replacement technique was used to collect samples. Subjects with the history of endocrinopathies (pituitary dysfunction, Cushing's syndrome and hypothyroidism) and history of medication such as phenothiazines, tricyclic antidepressant, anticonvulsants, and steroids were excluded from the study. 

Questionnaire was completed regarding complete demographic information (name, age, sex, address, education, socioeconomic status). Information regarding family history of obesity was obtained by drawing three-generation pedigrees and confirmation by two independent sources. Complete general physical examination was carried out. 

Subjects were further divided into 2 groups: less than or equal to 18 years (group 1) and greater than 18 years (group 2). In subjects >18 years, obesity was defined as BMI ≥30, and those with BMI <25 were categorized as nonobese [30]. Children and adolescents ≤18 years of age were divided into obese >95th percentile and nonobese 5th–85th percentile groups according to Center for Disease Control and Prevention (CDC) BMI for age growth charts [31]. 

### 2.2. Measurement of Anthropometric Parameters

Body weight, height, waist and hip circumference, systolic blood pressure (SBP), and diastolic blood pressure (DBP) were measured according to the standard procedures [32]. Body height was measured using a wall-mounted stadiometer, and body weight was measured using digital scale. BMI of each subject was calculated. Waist circumference was measured just above navel midway between the lower margin of the last rib and iliac crest to the nearest 0.1 cm. Hip circumference was taken as the maximal circumference over the buttocks. Waist-to-hip ratio was calculated from the values of waist and hip circumference. BP was measured twice from the right arm of the subject in a sitting position using a standard mercury sphygmomanometer.

### 2.3. Measurement of Metabolic Parameters

Blood samples were drawn after an overnight fast of 8–12 hours. Fasting blood glucose (FBG) levels were determined by the glucose oxidase method using HumaStar 180 chemistry analyzer (Human, Wiesbaden, Germany). Concentrations of plasma insulin, leptin, and leptin receptors were determined by ELISA using commercial kit with an automated EIA analyzer (Bio-Rad Laboratories, Hercules, CA, USA). FBG and fasting insulin levels were used to measure homeostasis model assessment of insulin resistance (HOMA-IR) calculated by the following formula: 

HOMA-IR = Fasting insulin (*μ*IU/mL) × Fasting glucose (mmol/L)/22.5 [33].

### 2.4. DNA Extraction and Genotyping

Genomic DNA was extracted from whole blood using genomic DNA purification kit (Fermentas, USA). Genotyping of rs9939609 polymorphism at *FTO* locus was carried out by polymerase chain reaction-restriction fragment length polymorphism assay (PCR-RFLP). A DNA fragment containing rs9939609 polymorphism was amplified using specific primers (forward primer sequence: AACTGGCTCTTGAATGAAATAGGATTCAGA and reverse primer sequence: AGAGTAACAGAGACTATCCAAGTGCAGTAC). The PCR was carried out using thermocycler (Icycler 5, BioRad, USA) according to the optimized conditions. In 25 *μ*L reaction, PCR components comprised of 50 ng DNA, 1X Taq buffer, 2 mM MgCl_2_, 200 *μ*M of each dNTP, 10 *ρ*mol of each primer, and 1U *Taq* DNA polymerase. Thermal cycling was performed as follows: initial denaturation at 95°C for 4 min, followed by 35 cycles of denaturation at 94°C for 30 sec, annealing at 58°C for 30 sec and extension at 72°C for 1 min, and then a final extension step at 72°C for 10 min.

Amplified products were digested with *ScaI* restriction enzyme (Favorgen, Taiwan) to analyze for polymorphism by RFLP assay.

### 2.5. Statistical Analysis

The data were analyzed using Statistical Package for Social Sciences (SPSS Inc. Chicago, IL, USA, version 17.0). Quantitative variables were expressed as mean ± standard error (SE). Student's *t*-test was applied to observe the differences between case and control groups. The whole data were stratified according to age and sex in subgroups. 

Hardy Weinberg equilibrium test (HWE) was applied to determine the variation in distribution of alleles and genotypes within the concerned population. Allelic frequencies were calculated by gene counting. Chi-square (*χ* ^2^) test was used to determine the significant differences of genotype and allelic frequencies between obese and nonobese groups. Association of rs9939609 variant with obesity was determined by Pearson Chi-square using codominant, dominant, and recessive models. Odds ratio (OR) and 95% confidence interval (CI) were calculated to determine the risk of obesity associated with the risk allele. The association of *FTO* rs9939609 variant with anthropometric and metabolic traits was determined using General Linear Model (GLM) assuming codominant, dominant, and recessive genetic models. The genotypes were coded as (0, 1, 2) in codominant model, (0, 1) in dominant model and (1, 0) in recessive model corresponding to the number of copies of risk allele. Analysis of variance (ANOVA), Tukey post hoc analysis and *t*-test were applied to test the differences of obesity-related anthropometric and metabolic traits across genotypes of rs9939609 variant adjusted for age and sex. Bonferroni adjustment was carried out for multiple comparisons. A *P* value of <0.05 was considered statistically significant. 

## 3. Results

Characteristics of study population are given in Table 1. Anthropometric and metabolic characteristics are summarized in Table 2. The frequency of minor A allele was 0.19. The genotypes of rs9939609 variant were in Hardy-Weinberg equilibrium (*P* > 0.05) in both obese and nonobese groups.

We compared the distribution of genotype and allele frequencies for rs9939609 variant using Chi-square test in obese and nonobese subjects. The frequency of A allele was higher in obese group as compared to nonobese group (*P* < 0.05) with higher frequency in obese females as compared to nonobese females (*P* < 0.05) (Table 3). 

We stratified the whole data according to age in two groups: ≤18 years and >18 years. Frequency of A allele was significantly higher in >18 years obese group (*P* < 0.05) as compared to nonobese subjects, whereas no significant difference was observed in children and adolescents (Table 3). There was significantly higher frequency of AA and AT genotypes and A allele (*P* < 0.05) in adult obese females (>18 years) as compared to nonobese, whereas no significant difference was observed in male subjects (>18 years) (Table 3). The above results revealed major age and gender differences in genotype and allele frequencies of rs9939609 variant, with higher frequency of AA genotype and obesity risk A allele in adult obese female subjects but not in children or males (Table 3).

### 3.1. Association of *FTO* Variant with Obesity

We found significant association of rs9939609 variant with obesity in a dominant model (*P* < 0.05, 95% CI = 1.0–2.6) (Table 3). Association of rs9939609 variant with obesity was also examined in >18 years and ≤18 years groups after stratification; association was found only in the group with >18 years adult subjects. The data were further analyzed for gender differences; *FTO* variant associated significantly with obesity (*P* < 0.01, 95% CI = 1.6–9.2) only in adult females (>18 years old). *FTO* minor allele (A allele) increased the risk of obesity by 2.8 times (95% CI = 1.3–6.0, *P* < 0.05) in adult females (Table 3).

### 3.2. Association of *FTO* Variant with Anthropometric Parameters and Metabolic Traits

 General linear model (GLM) multivariate analysis of the subgroup with adult female subjects using age as a covariate in a dominant model revealed significant association of rs9939609 variant with body weight, BMI, waist and hip circumference, WHR, BP, FBG, HOMA-IR and leptin receptor levels (*P* < 0.05 for all traits) but not with height, plasma insulin, and leptin levels (*P* > 0.05). The associations remained significant after adjusting the data for BMI; moreover, the association of rs9939609 variant with insulin and leptin became significant after adjusting the data for BMI (Table 4).

In adult females when anthropometric and metabolic parameters were compared between carriers of AA/AT genotype and TT carriers by *t*-test, significant (*P* < 0.05 for all traits) increase in the BMI, waist and hip circumference, SBP, FBG, HOMA-IR, and low plasma leptin receptor levels was observed in carriers of A allele compared to TT homozygotes. However, no significant difference (*P* > 0.05) was observed in WHR, DBP, insulin and leptin levels between carriers of A allele and TT homozygotes (Table 4).

## 4. Discussion

Studies analyzing the association of rs9939609 variant with obesity and BMI in Asian populations have revealed controversial results [14, 17, 34, 35]. Our study on Pakistanis reports significant association of rs9939609 variant with obesity, body weight, BMI, waist and hip circumference, and WHR but not with height in adult females. This indicates the role of this variant in fat deposition and predisposition to obesity after adolescence. These results are in agreement with the report of a meta-analysis that described significant association of *FTO* variant with BMI in Asian adults [36]. Association of *FTO* variant with BMI and obesity has been reported in East Asians [35, 37] and with T2D in Indians [24, 38]. Our study reported significant association of *FTO* variant with BMI and obesity in Pakistani females <45 years of age. Similar association of *FTO* variant with BMI and T2D has been reported in Pakistani subjects >40 years of age [39]. In addition, our study reported significant association of rs9939609 variant with BP, FBG, HOMA-IR, and leptin receptor levels in adult females. These associations remained significant after adjusting the BMI; however, the association of rs9939609 variant with increased plasma insulin and leptin levels became significant when BMI was adjusted. This indicates that the association is only partially affected by BMI in this population as compared to Europeans. 

In contrast to the European population with minor allele frequency (MAF) of 0.45 [6], we found substantially low MAF (0.19). Likewise, other studies in Asian populations also reported low MAF (0.1–0.20) [23, 40]. These differences in MAFs from different studies might be attributable to differences in BMI standards, ethnic groups, sample sizes, sample collection, and environmental exposure. Despite low MAF in our study, we observed significant differences in genotype and allele frequency between obese and nonobese subjects. Moreover, we also observed major age and gender differences as association of *FTO* variant with BMI and obesity was found in adult females only and not in girls. Likewise, Jacobsson et al. also reported gender-related development of obesity and association of rs9939609 variant with obesity among girls [25]. However, initial studies in European populations reported no gender differences in association of *FTO* common variant with obesity [6]. No association was observed in males in our study; however, association of rs9939609 variant with BMI has been reported in Danish men [41]. Lack of association in adult males may be due to variation in the body fat distribution between males and females. Our study reported no association of this variant with obesity or BMI in children and adolescents ≤18 years of age; in contrast to this, various studies have reported the association of rs9939609 variant with obesity in children [6, 11, 18, 23].

## 5. Conclusions

The association of rs9939609 variant of *FTO* gene in adult Pakistani females with BMI and obesity and with measures of body fat distribution such as waist and hip circumference and a ratio of waist-to-hip circumference suggest the role of this polymorphism in fat deposition and a predisposing factor for obesity. Moreover, association of rs9939609 variant of *FTO* gene in adult Pakistani females with higher FBG and plasma leptin levels indicates that this polymorphism may disturb metabolism in adulthood and predispose to T2D. Although rs9939609 variant of *FTO* gene is consistently associated with obesity in worldwide populations, sex and age dependence of such association in Pakistani population is a question worth consideration in future studies.

## Figures and Tables

**Table 1:** Characteristics of study population.

	Obese *n* = 239	Nonobese/control *n* = 155
Males	111 (46%)	70 (45%)
Females	128 (54%)	85 (55%)
>18 years	161 (67%)	97 (63%)
>18 years males	64 (40%)	52 (54%)
>18 years females	97 (60%)	45 (46%)
≤18 years	78 (33%)	58 (37%)
≤18 years males	47 (60%)	18 (31%)
≤18 years females	31 (40%)	40 (69%)
Acanthosis nigricans	102 (43%)	5 (3%)
Hypertension	2 (18%)	0
Impaired fasting glucose	2 (33%)	5 (3%)
Cardiovascular disease	10 (4%)	0
Family history of obesity	184 (77%)	48 (31%)

**Table 2:** Anthropometric and biochemical parameters of obese and nonobese subjects.

	Obese (*n* = 239)	Nonobese (*n* = 155)	*P* value
Age (years)	25.2 ± 0.7	23.9 ± 0.7	>0.05
Body weight (Kg)	84.8 ± 1.6	56.6 ± 1.0	<0.05*
Height (m)	1.5 ± 0.01	2.0 ± 0.4	>0.05
BMI (Kg/m^2^)	34.2 ± 0.4	21.1 ± 0.2	<0.05*
Waist circumference (cm)	102.8 ± 1.1	73.1 ± 1.1	<0.05*
Hip circumference (cm)	112 ± 1.1	87.5 ± 1.2	<0.05*
Waist-to-hip ratio	0.9 ± 0.004	0.8 ± 0.01	<0.05*
Systolic blood pressure (mmHg)	126.4 ± 4.4	106 ± 0.9	<0.05*
Diastolic blood pressure (mmHg)	82.7 ± 0.6	71.2 ± 0.6	<0.05*
Fasting blood glucose (mg/dL)	101.4 ± 2.0	90.5 ± 1.8	<0.05*
Insulin (*µ*IU/mL)	23.0 ± 1.8	5.8 ± 0.3	<0.05*
HOMA-IR	5.2 ± 0.5	1.3 ± 0.08	<0.05*
Leptin (ng/mL)	33.2 ± 1.6	8.3 ± 0.7	<0.05*
Leptin receptor (ng/mL)	16.3 ± 0.9	19.7 ± 0.7	<0.05*

Continuous variables are presented as mean ± SEM and were compared by *t*-test. *P < 0.05 was considered as significant.

**Table 3:** Genotype and allele distribution of the rs9939609 variant of *FTO *gene in obese and non-obese subjects.

Genotype	All subjects		*χ* ^2^ /P/OR	Females		*χ* ^2^/P/OR	Males		*χ* ^2^/P/OR	>18 years		*χ* ^2^/P/OR	Females > 18 years		*χ* ^2^/P/OR	Males > 18 years		*χ* ^2^/P/OR	≤18 years		*χ* ^2^/P/OR
	Obese *n* = 239	Nonobese *n* = 130		Obese *n* = 128	Nonobese *n* = 71		Obese *n* = 111	Nonobese *n* = 59		Obese *n* = 161	Nonobese *n* = 85		Obese *n* = 97	Nonobese *n* = 41		Obese *n* = 64	Nonobese *n* = 44		Obese *n* = 78	Nonobese *n* = 45	
	Codominant model																				
AA	6 (2%)	2 (1%)		4 (3%)	1 (1%)		2 (1.8%)	1 (1.6%)		4 (2%)	1 (1%)		3 (3%)	1 (2%)		1 (1.5%)	0		2 (2.6%)	0	
TA	87 (36%)	34 (26%)	4.6/0.09	52 (41%)	16 (23%)	**7.7/0.02**	35 (31.5%)	18 (30.5%)	0.02/0.9	61 (38%)	20 (24%)	1.7/0.4	44 (45%)	7 (18%)	**10.2/0.005**	17 (26.5%)	13 (29.5%)	0.7/0.6	26 (33.3%)	15 (33.3%)	1.1/0.5
TT	146 (61%)	94 (72%)		72 (56%)	54 (76%)		74 (66.6%)	40 (67.7%)		96 (60%)	64 (75%)		50 (51%)	33 (80%)		46 (71.8%)	31 (70%)		50 (64%)	30 (66.6%)	

	Dominant model																				
A/A + A/T	93 (39%)	36 (27%)		56 (44%)	17 (24%)		37 (33%)	19 (32.2%)		65 (40%)	21 (25%)		47 (48%)	8 (20%)		18 (28%)	13 (29.5%)		28 (36%)	15 (33.3%)	
T/T	146 (61%)	94 (72%)	**4.6/0.03/1.6**	72 (56%)	54 (76%)	**7.7/0.005/2.4**	74 (66.6%)	40 (67.7%)	0.02/0.8/1.0	96 (60%)	64 (75%)	**6/0.01/2**	50 (51%)	33 (80%)	**10.0/0.001/3.8**	46 (72%)	31 (70%)	0.02/0.8/0.9	50 (64%)	30 (66.6%)	0.08/0.7/1.1

	Recessive model																				
A/A	6 (2%)	2 (1%)		4 (3%)	1 (2%)		2 (1.8%)	1 (1.6%)		4 (2%)	1 (1%)		3 (3%)	1 (2%)		1 (1.5%)	0		2 (2.6%)	0	
A/T + T/T	233 (97%)	128 (98%)	0.3/0.5/1.6	124 (97%)	70 (98%)	0.5/0.4/2.2	109 (98%)	58 (98%)	0/1/1.0	157 (98%)	84 (99%)	0.3/0.5/2.1	94 (97%)	40 (98%)	0.04/0.8/1.2	63 (98.4%)	44 (100%)	0.6/0.4	76 (97.4%)	45 (100%)	0/0.5

	Alleles																				
A	99 (21%)	38 (15%)		60 (31%)	[18 (13%)]		39 (17.5%)	20 (16.9%)		69 (21%)	22 (13%)		50 (26%)	9 (11%)		19 (14.8%)	13 (14.8)		30 (19.3%)	15 (16.6%)	
T	379 (79%)	222 (85%)	**4.1/0.04/1.5**	131 (69%)	124 (87%)	**15.9/<0.0001/3.1**	183 (82.5%)	98 (83%)	0.02/0.8/1.0	253 (79%)	148 (87%)	**5.3/0.02/1.8**	144 (74%)	73 (89%)	**7.5/0.006/2.8**	109 (85%)	75 (85.2%)	0/1/0.005	126 (80.7%)	75 (83.3%)	0.2/0.6/1.1

T = Wild; A = polymorphic.

**Table 4:** Association of *FTO* rs9939609 variant with anthropometric and metabolic traits in >18 years old female subjects using dominant model.

	TT *n* = 83	TA/AA *n* = 55	GLM *P* value	BMI adjusted *P* value	*t*-test *P* value
Body weight (Kg)	77.5 ± 2.4	90.5 ± 2.7	<0.001*	<0.001*	0.04*
Height (m)	1.5 ± 0.01	1.5 ± 0.01	0.1	0.1	0.03*
BMI (Kg/m^2^)	31.1 ± 0.9	35.7 ± 0.9	<0.001*	<0.001*	0.01*
Waist circumference (cm)	94.6 ± 2.3	105 ± 2.0	<0.001*	<0.001*	0.01*
Hip (cm)	112 ± 2.0	119 ± 1.9	<0.001*	<0.001*	0.02*
WHR	0.8 ± 0.008	0.8 ± 0.01	<0.001*	<0.001*	0.06
SBP (mmHg)	116 ± 1.5	124 ± 1.6	<0.001*	<0.001*	0.003*
DBP (mmHg)	80.1 ± 1.1	83.1 ± 1.1	<0.001*	0.001*	0.16
FBS (mg/dL)	93.8 ± 2.4	106 ± 4.8	<0.001*	0.001*	0.008*
Insulin (*µ*IU/mL)	13.0 ± 1.3	19.4 ± 2.8	0.2	0.001*	0.20
HOMA-IR	2.9 ± 0.3	5.2 ± 0.7	0.03*	<0.001*	0.02*
Leptin (ng/mL)	27.5 ± 2.3	32.7 ± 3.2	0.3	<0.001*	0.18
Leptin receptor (ng/mL)	18.9 ± 0.9	15.7 ± 0.6	0.03*	<0.001*	0.03*

Data are presented as mean ± SEM and were compared by *t*-test and GLM after adjusting the data for BMI. *P < 0.05 was considered as significant.
